# Mid‐Term Follow‐Up of Acetabular Revision Arthroplasty Using Jumbo Cups

**DOI:** 10.1111/os.12522

**Published:** 2019-09-24

**Authors:** Ji Zhang, Yong Huang, Baochun Zhou, Yixin Zhou

**Affiliations:** ^1^ Department of Orthopaedic Surgery Beijing Jishuitan Hospital, Fourth Clinical College of Peking University Beijing China

**Keywords:** Acetabular revision, Hip revision, Jumbo cup, Center of rotation

## Abstract

**Objective:**

To evaluate the mid‐term clinical and radiographic results of revision total hip arthroplasty (THA) using jumbo cups in Chinese patients.

**Methods:**

We retrospectively studied 61 patients (63 hips; 29 men [30 hips], 32 women [33 hips]) who underwent acetabular revision with jumbo cups between January 2001 and April 2016 at our institution. The mean age at the index operation was 59.4 ± 11.4 years. The mean body mass index of the patients was 24.9 ± 3.8 kg/m^2^. Clinical evaluation was determined using the Harris hip score preoperatively and at follow up. Major complications (including instability, sepsis, and revision of the femoral or acetabular component) were recorded. Radiographic measurements included inclination and anteversion angles of the acetabular components, and the vertical and horizontal distances of the centers of rotation (V‐COR and H‐COR, respectively). In the 42 patients with normal contralateral hip joints, the postoperative V‐COR and H‐COR were compared between right and left sides. Their improvement in leg‐length discrepancy (LLD) after revision THA was also evaluated. Cup survival was evaluated using the Kaplan–Meier analysis. Clinical and radiographic outcomes were analyzed.

**Results:**

Mean follow up was 5.7 years (2–16 years). At the latest follow‐up, the average Harris hip score (preoperative *vs* postoperative values) had improved from 46 to 83 (*P* < 0.001). No acetabular component was radiographically defined as loosened. Four hips (6.3%) had major complications: one hip was revised because of periprosthetic infection (at 3 months); one underwent femoral open reduction and internal fixation (with implant retention) because of a periprosthetic femoral fracture (at 13 months); one operated hip developed a deep infection (at 2.5 years), which was treated with antibiotics; one hip experienced recurrent dislocation (at 4.5 years). The average cup inclination angle was 40.8° ± 6.8° and the average anteversion angle was 14.9° ± 6.6°. Average V‐COR decreased from 29.7 ± 10.4 mm to 22.3 ± 7.6 mm (*P* < 0.001). The average postoperative H‐COR was 29.5 ± 3.7 mm compared with 30.8 ± 6.6 mm preoperatively (*P* = 0.145). Among the 42 patients with normal contralateral hips, the average postoperative V‐COR were 22.2 ± 8.3 mm (operated side) and 14.0 ± 3.7 mm (contralateral side) (*P* < 0.001). LLD improved from −16.8 ± 17.1 mm to −5.6 ± 11.8 mm (*P* < 0.001). When failure was defined as any reoperation involving the hip, the mean 16‐year hip survival was 96.8% (95% confidence interval [CI] 87.9%–99.2%). When defined as any hip reoperation or major complication, it was 92.7% (95%CI 81.2%–97.2%).

**Conclusion:**

Use of jumbo cups for revision THA resulted in excellent mid‐term cup survival and helped restore the COR.

## Introduction

Good long‐term results have been achieved with cementless porous‐coated cups during acetabular revision^1^, ^2^, ^3^. Porous‐coated cups combined with the use of special techniques are available for addressing even large bone defects. The main techniques include placing: (i) a cup in a superior position; (ii) a cup composed of structured allograft; (iii) a cup with trabecular metal augments; (iv) a cup of jumbo dimensions^4^, ^5^, ^6^, ^7^.

Using a cementless jumbo cup is a straightforward, effective technique for treating extensive bone defects during acetabular revision. The jumbo cup technique has some advantages, including the relative simplicity of the procedure, provision of maximum surface contact between the component and the host bone, reduced need for bone grafting, and possible normalization of the hip's center of rotation (COR).

Some authors have reported encouraging clinical results with jumbo cups. Dearborn and Harris reported the mid‐term clinical and radiographic results from a group of 15 patients using jumbo cups during revision total hip arthroplasty (THA). After an average 7‐year (overall 5.0–10.3 years) follow up, the mean final Harris hip score was 86 points (45–100 points). In all, 7 hips were judged excellent, 6 good, 1 fair, and 1 poor. No acetabular component was revised for aseptic loosening. None had migrated or was loose^8^. In the study by Patel *et al*., 36 hips were revised with jumbo cups combined with morcellized allograft or bulk allograft material. After an average 10‐year (overall 6–14 years) follow up, only two acetabular components had been revised because of aseptic loosening. The 14‐year survival rate was 92%^9^. However, most of these reports were from North American. We found only one study, by Fan *et al*., that focused on an Asian population^10^. They reported similar clinical and radiographic results from 47 hips revised with jumbo cups, among which 44 (93.6%) had acetabular components that were well‐fixed after a mean follow up of 65 (range 48–84) months. The 5‐year survival rate of these jumbo cups was 94.5%, estimated using the Kaplan–Meier analysis. In addition, the average COR was improved from 31 mm preoperatively to 27 mm postoperatively. We therefore aimed to determine if we could achieve results in Asian patients that were similar to those achieved in Western countries. Hence, the purpose of this study was to evaluate mid‐term clinical and radiographic results of revision THA during which jumbo cups had been implanted in Chinese patients.

## Patients and Methods

### *Ethics Approval*

Approval of the study was obtained from the Beijing Jishuitan Hospital institutional review board.

### *Patient Data*

Inclusion criteria: (i) patients with a failed hip arthroplasty (e.g. aseptic loosening, sepsis, periprosthetic fracture, and wear); (ii) patients who had undergone hip revision with cementless jumbo cups (the cup size used in the hip revision was ≥64 mm in male patients and ≥ 60 mm in female patients); (iii) the main evaluation indicators included survivorship, Harris hip score, complications, inclination and anteversion angles of the cups, vertical and horizontal distances of the COR, and leg‐length discrepancy (LLD), and (iv) a retrospective cohort study.

Exclusion criteria: (i) patients whose acetabula were reconstructed with cemented acetabular components; (ii) patients whose acetabula were reconstructed with a cementless acetabular component combined with a structural allograft or metal augment; (iii) patients whose acetabular components were placed in a superior position; and (iv) postoperative follow‐up time was less than 2 years.

We reviewed all 791 revision THA that had been performed at our institution with cementless acetabular cups between January 2001 and April 2016. Patients whose acetabula were reconstructed with cementless jumbo cups were included. The Mayo Clinic's definition of a “jumbo cup” is one that is ≥66 mm for men and ≥62 mm for women (each being 10 mm larger than the mean cup diameter usually used for primary THA)^4^. In contrast, Fan *et al*. stated that the diameter of the jumbo cups for Asians should be 2 mm smaller than those commonly used in Western countries^10^. Therefore, we calculated the mean cup size for >12 000 primary THA at our institution, which yielded sizes of 54.7 mm for men and 49.5 mm for women. We then modified the “jumbo” cup sizes to 64 mm for men and 60 mm for women to accommodate our Chinese population.

According to the criteria mentioned above, 73 revision THA in 71 patients were classified as having been performed using jumbo cups. Among them, 8 patients (8 hips) were lost to follow up, and 2 patients (2 hips) died after 2 and 8 years, respectively, with their implants still in place. The causes of death were unrelated to the surgery. The final study group (whose clinical and radiographic evaluations were complete) included the remaining 61 patients (63 hips), with a mean follow up of 5.7 years (range 2–16 years). Among them, 29 (30 hips) were men and 32 (33 hips) were women. The mean age at the time of the index operation was 59.4 ± 11.4 years. The mean body mass index of the patients was 24.9 ± 3.8 kg/m^2^. The indications for revision were aseptic loosening (*n* = 55), second stage of a two‐stage revision procedure due to sepsis (*n* = 6), and wear and osteolysis (*n* = 2). According to the classification system proposed by Paprosky *et al*.^11^, the bone deficiencies of the 63 patients were classified as type IIA in 16, type IIB in 9, type IIC in 24, type IIIA in 8, and type IIIB in 6.

### *Surgical Technique*

All revision arthroplasties were performed by senior surgeons at our institution with the patients under general or epidural anesthesia. Patients were placed in a lateral decubitus position. A posterolateral approach was applied in all cases. Extended trochanteric osteotomy was used in case of cement removal on the femoral side. After removing the failed cup, the bony rim of the acetabulum was exposed, and fibrous tissues in the acetabulum were debrided. The bone defects of the acetabulum were evaluated before acetabular reaming. Most patients had a distorted acetabular rim with the superoinferior diameter much greater than the anteroposterior diameter because of superior migration of the failed cup. In some cases of severe bone defects, the acetabular rims were not intact.

The size of the first reamer was approximately 4–6 mm smaller than the size of the failed cup. Thereafter, the size of the reamer was increased gradually until it engaged the anterior wall, the posterior wall, and the superior rim. The acetabular shell was under‐reamed 1–2 mm to the planned cup size, depending on the quality of the residual bone. The cup was then hammered in. Initial fixation was supplemented with multiple screws. In patients with acetabular protrusion, morcellized allograft was applied before implanting the cup.

Four types of cementless acetabular component were used for these reconstructions: Trabecular Metal cup (Zimmer, Warsaw, IN, USA) was used in 57 cases, T.O.P. cup (Waldemar Link, Hamburg, Germany) in 3 cases, Pinnacle cup (Depuy, Warsaw, IN, USA) in 2 cases, and Secur‐fit cup (Osteonic, Allendale, NJ, USA) in 1 case. The sizes of the cups and femoral heads used in the revision arthroplasties are shown in Tables 1 and 2.

**Table 1:** Diameters of the acetabular components used for revision arthroplasty

Diameter of acetabular component (mm)	Number of hips
60	17 (27.0%)
62	10 (15.9%)
64	22 (34.9%)
66	10 (15.9%)
68	2 (3.2%)
70	2 (3.2%)

**Table 2:** Diameters of the femoral head used for revision arthroplasty

Diameter of femoral head (mm)	Number of hips
28	16 (25.4%)
32	44 (69.8%)
36	3 (4.8%)

### *Outcome Measures*

#### *Harris Hip Score*

Patients were requested to return for clinical evaluation and radiography at 3, 6, and 12 months and then annually. Patients who were unable to return to our institution were followed by telephone calls and were asked to have radiographs prepared locally and sent to us. Clinical evaluation was determined using the Harris hip score^12^ preoperatively and at follow‐up. The patients were excluded from clinical evaluation if major complications were found to have occurred at the follow‐up.

The Harris hip score is a 100‐point scale that comprises the subcategories of pain (44 points), function (47 points), range of movement (5 points), and absence of deformity (4 points). The hips were judged as excellent at scores of 90–100 points, good at 80–89 points, fair at 70–79, and poor at <70 points.

#### *Complications*

Major complications (instability, sepsis, and revision of the femoral or acetabular component) were recorded. Any reoperations related to the operated hips were also recorded.

#### *Radiological Loosening*

Component loosening and radiolucent lines were identified by one of the senior authors. Acetabular loosening was defined as >2 mm of component migration, screw fracture, or the presence of circumferential radiolucent lines^13^. Radiolucent lines at the prosthesis–bone interface were recorded using the three zones described by DeLee and Charnley^14^.

#### *Inclination and Anteversion Angles of the Cup*

The radiographic measurements were performed by one observer and were obtained from anteroposterior pelvic radiographs using the Materialise Interactive Medical Image Control System (Mimics, Version 10.01; Materialise, Leuven, Belgium). The teardrop and inter‐teardrop lines were used as landmarks for measurements because they are discrete anatomic structures^15^. Measurements of the inclination angles of the acetabular components were referenced from the inter‐teardrop line. We used the method described by Lewinnek *et al*.^16^ to measure the anteversion angle of the cups. After drawing an ellipse along the acetabular cup rim, we measured the distances of the short and long axes of the ellipse. The anteversion angle equals the arcsin (short axis/long axis).

#### *Vertical and Horizontal Distances of the Cup*

**Figure 1:**
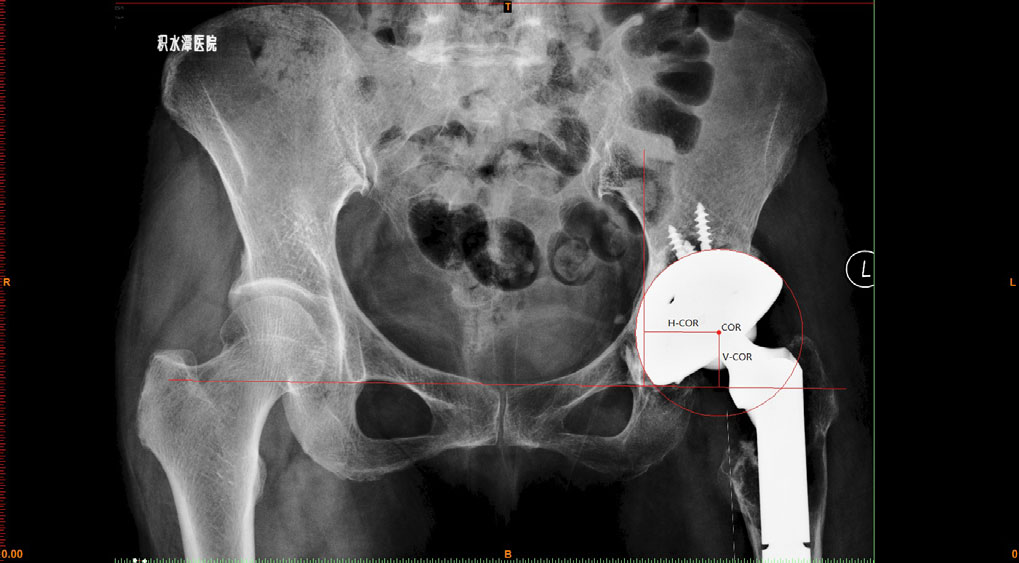
Radiographic measurements using Mimics 10.0 software on an anteroposterior (AP) pelvic radiograph. COR, center of rotation; H‐COR, horizontal distance of the COR; V‐COR, vertical distance of the COR.

The position of the cup was defined by the vertical and horizontal distances of the COR (V‐COR and H‐COR, respectively) in relation to the teardrop, which was described by Russotti and Harris^17^. The V‐COR was measured as the distance between the COR and the inter‐teardrop line. The H‐COR was measured as the distance between the COR and the perpendicular line through the inferior point of the teardrop. We compared the changes in V‐COR and H‐COR before and after surgery. For the 42 patients whose contralateral hip joints were normal, we compared the V‐COR and H‐COR on the right and left sides postoperatively (Fig. 1).

#### *Leg‐Length Discrepancy*

We also evaluated the improvement in LLD after revision THA in the 42 patients with a normal contralateral hip joint. The LLD was measured as the difference in the perpendicular distances between the tip of the lesser trochanter and the inter‐teardrop line on both sides. A positive value indicated that the operated limb was longer than that the contralateral limb, and a negative value indicated the opposite^18^.

#### *Data Correction*

To calculate the magnification of each radiograph, the diameter of the cup was measured on the radiograph and divided by its diameter. All distance parameters were adjusted according to the magnification of each image.

### *Statistical Analysis*

Statistical analyses were performed using SPSS 11.0 (SPSS, Chicago, IL, USA). The paired *t*‐test was used to assess changes in the Harris hip score, V‐COR and H‐COR, and the LLD before and after revision operations. A value of *P* < 0.05 indicated statistical significance. Component survival was calculated using the Kaplan–Meier method. Kaplan–Meier cup survival analysis was performed using Stata 12.0 software (Stata, College Station, TX, USA), with the endpoint defined as either (i) any reoperation or (ii) reoperation and major complications (e.g. periprosthetic infection or recurrent dislocation).

## Results

### *Harris Hip Score*

**Figure 2:**
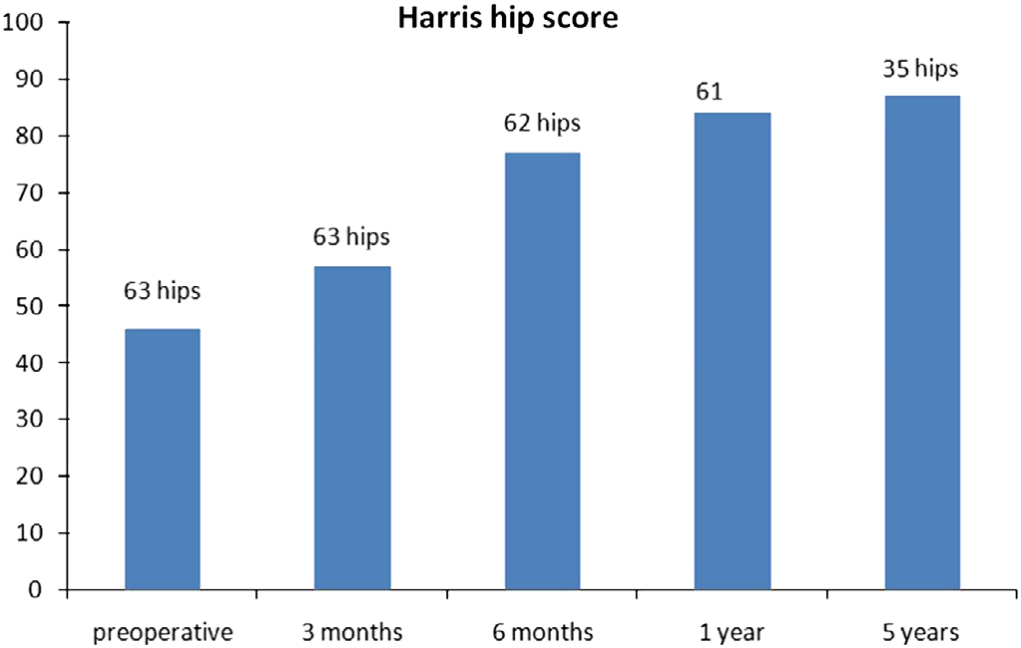
Average preoperative and postoperative Harris hip score.

The average preoperative Harris hip score was 46. The average postoperative Harris hip scores were 57, 77, 84, and 87, respectively, at 3 months (63 hips), 6 months (62 hips), 1 year (61 hips), and 5 years (35 hips) (Fig. 2). At the latest follow‐up, the average Harris hip score was 83 for the 59 patients who had no major complications, which was significantly better than that preoperatively (*P* < 0.001, t = −14.0). Results were rated excellent in 17 hips, good in 25, fair in 14, and poor in 3.

### *Reoperation and Complications*

**Figure 3:**
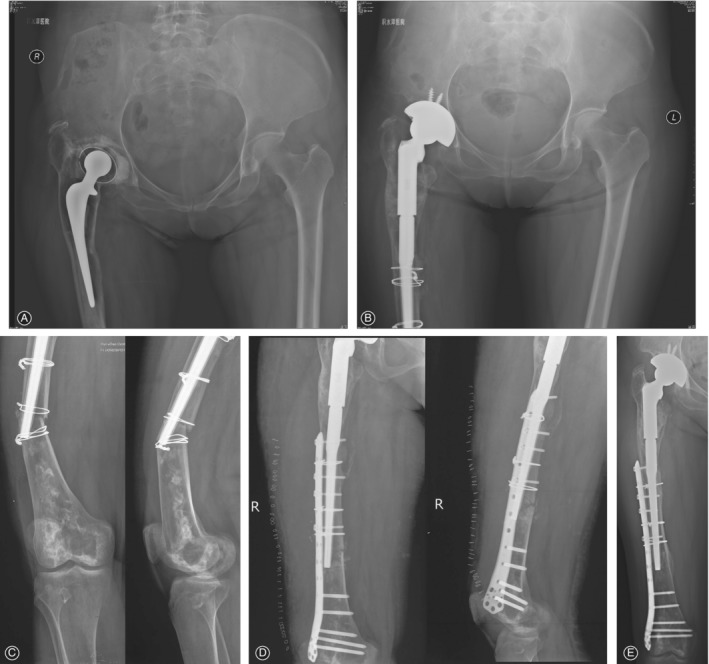
(A) Preoperative anteroposterior (AP) radiograph of the right hip in a patient requiring revision because of aseptic loosening. (B) AP radiograph 2 months after revision arthroplasty with a 64‐mm diameter cementless acetabular component and a modular revision stem. (C) The patient experienced a Vancouver B1 periprosthetic femoral fracture 13 months later. (D) The patient was treated with open reduction and internal fixation (ORIF) of the femur. (E) AP radiograph 3.5 years after the ORIF.

**Figure 4:**
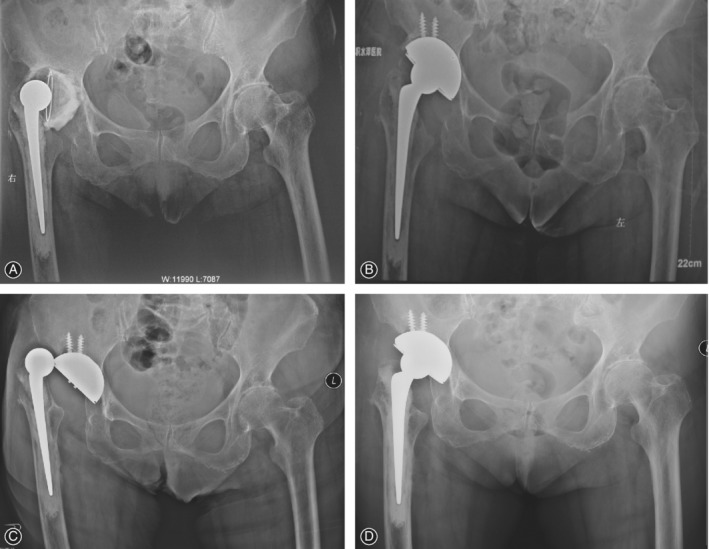
(A) Preoperative anteroposterior (AP) radiograph of the right hip in an 81‐year‐old woman requiring revision because of aseptic loosening. (B) AP radiograph right after revision arthroplasty with a 62‐mm diameter cementless acetabular component and a cemented stem. (C) The patient experienced recurrent dislocation 4.5 years later and was treated with closed reduction. (D) The components were well‐fixed at the 8.5‐year follow up.

Four patients (four hips, 6.3%) had major complications. One patient (one hip) underwent revision for periprosthetic infection after 3 months. One patient (one hip) underwent open reduction and internal fixation of the femur because of a periprosthetic femoral fracture after 13 months, with retention of his implant (Fig. 3). One patient (one hip) was diagnosed with a deep infection in the operated hip after 2.5 years. Because of his poor physical condition, he was treated with antibiotics. His implant remained *in situ*. One patient (one hip) experienced recurrent dislocation after 4.5 years. She refused revision because of her advanced age and poor health (Fig. 4).

### *Radiological Loosening*

**Figure 5:**
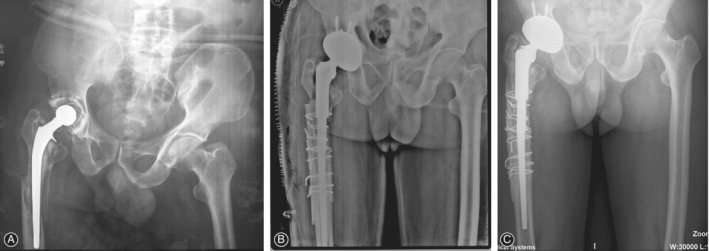
(A) Preoperative anteroposterior (AP) radiograph of the left hip in a patient requiring revision because of aseptic loosening of the cup and periprosthetic fracture of the stem. (B) AP radiograph right after revision arthroplasty with a 64‐mm diameter cementless acetabular component and a modular revision stem. (C) AP radiograph 13.5 years after the index revision.

Among the 61 patients (63 hips) with complete follow up, none of the acetabular components was defined radiographically as loosened (Fig. 5). Nonprogressive radiolucent lines at the bone–acetabular component interface were detected in four hips (6.3%) at the latest follow‐up. Radiolucent lines were apparent in zone I in three hips and in zone II in one hip.

### *Inclination and Anteversion Angles of the Cup*

The average cup inclination angle was 40.8° ± 6.8°, and the average anteversion angle was 14.9° ± 6.6°.

### *Vertical and Horizontal Distances of the Cup*

The average postoperative V‐COR was 22.3 ± 7.6 mm compared with 29.7 ± 10.4 mm preoperatively (*P* < 0.001). The average postoperative H‐COR was 29.5 ± 3.7 mm compared with 30.8 ± 6.6 mm preoperatively (*P* = 0.145).

Among the 42 patients whose contralateral hips were normal, the average postoperative V‐COR was 22.2 ± 8.3 mm on the operated side and 14.0 ± 3.7 mm on the contralateral side (*P* < 0.001). The average postoperative H‐COR was 29.4 ± 3.8 mm on the operated side and 30.3 ± 3.3 mm on the contralateral side (*P* = 0.184).

### *Leg‐Length Discrepancy*

The LLD was improved from −16.8 ± 17.1 mm preoperatively to −5.6 ± 11.8 mm postoperatively (*P* < 0.001) (Table 3).

**Table 3:** Changes in V‐COR, H‐COR, and LLD after revision THA based on radiographic measurements

	Number	Mean	SD	*P*
V‐COR pre.	63	29.7	10.4	<0.001
V‐COR post.	63	22.3	7.6	
H‐COR pre.	63	30.8	6.6	0.145
H‐COR post.	63	29.5	3.7	
V‐COR post. (operated side)	42	22.2	8.3	<0.001
V‐COR post. (contralateral side)	42	14.0	3.7	
H‐COR post. (operated side)	42	29.4	3.8	0.184
H‐COR post. (contralateral side)	42	30.2	3.3	
LLD pre.	42	−16.8	17.1	<0.001
LLD post.	42	−5.6	11.8	

H‐COR, horizontal distance of the center of rotation; LLD, leg‐length discrepancy; pre., before the operation; post., after the operation; THA, total hip arthroplasty; V‐COR, vertical distance of the center of rotation

### *Component Survival*

**Figure 6:**
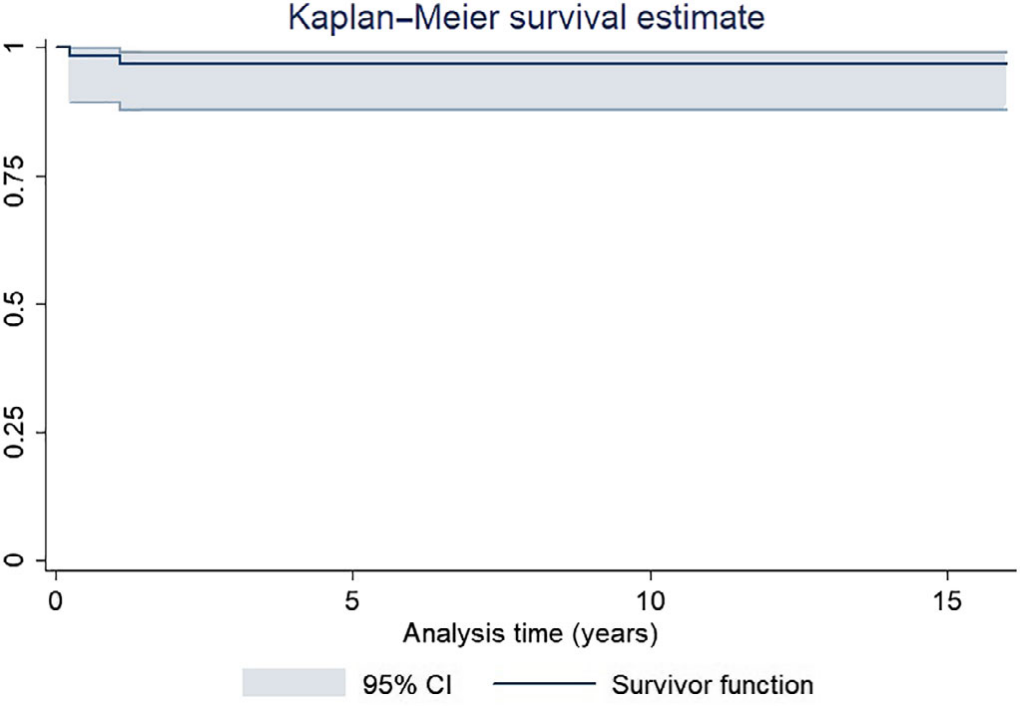
Kaplan–Meier analysis of cup survival after hip revisions using jumbo cups. Failure was defined as any reoperation involving the hip.

**Figure 7:**
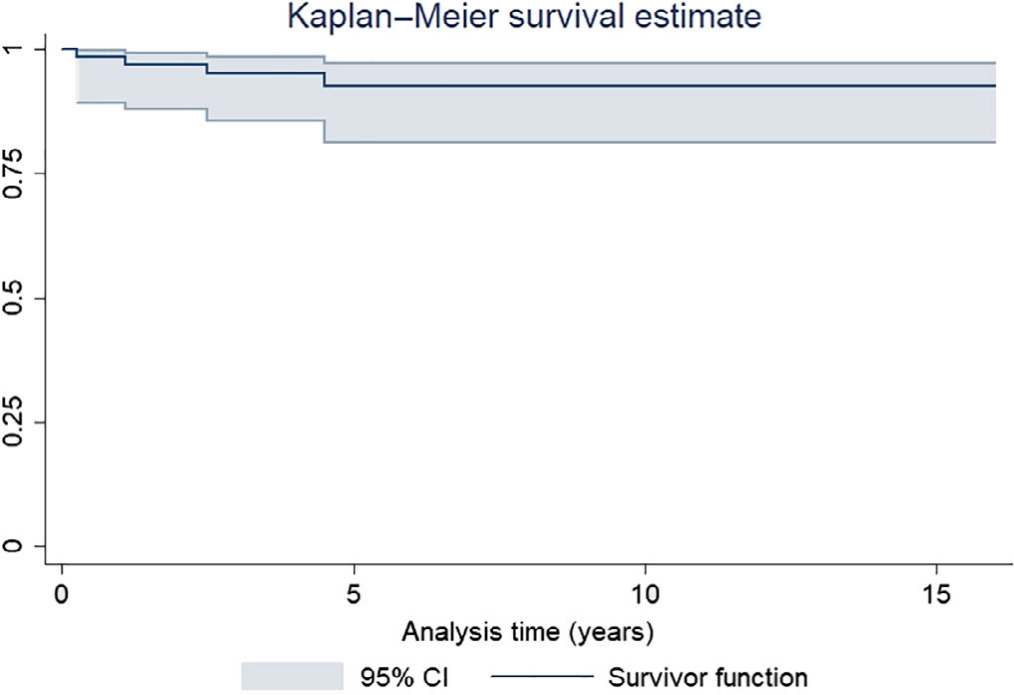
Kaplan–Meier analysis of cup survival after hip revisions using jumbo cups. Failure was defined as any hip reoperation or major complication.

When failure was defined as any reoperation involving the hip (Fig. 6), the 16‐year survival was 96.8% (95% CI 87.9–99.2). When failure was defined as any reoperation or major complication (Fig. 7), the 16‐year survival was 92.7% (95% CI 81.2–97.2).

## Discussion

The alternatives for reconstructing an acetabulum with large bone defects during revision THA include high hip center placement^19^, a structural allograft^20^, bi‐lobed oblong cups^21^, reconstruction cages^22^, tantalum augments^23^, and jumbo cup replacements. The use of a jumbo cup, compared with other methods, has some obvious advantage, including the relative simplicity of the procedure, provision of maximum surface contact between the component and the host's bone, reduced need for bone grafting, and possible normalization of the hip's COR^24^. Some authors have reported satisfactory results after the use of jumbo cups^4^, ^8^, ^9^, ^24^, ^25^. In the present study, no acetabular component showed aseptic loosening, and only 4 of 63 hips required reoperation or were associated with major complications.

Dearborn and Harris proposed a definition of “jumbo cup”^8^. According to their definition, a socket could be called a “jumbo cup” if its outside diameter was ≥66 mm. The definition proposed by Whaley *et al*.^4^, however, seems more reasonable because it differentiates between the sexes and is based on the average size of cups used for primary THA. Using Whaley and colleagues' method, Fan *et al*.^10^ assumed that the size of a “jumbo cup” of an Asian population should be ≥64 mm for men and ≥60 mm for women, which they determined by calculating the average size of acetabular cups used for primary THA at their institution. We echo the opinion of Fan *et al*. because we yielded a similar average size of acetabular cups to be used for primary THA at our institution.

Previous studies reported high dislocation rates (i.e. 9.3%–21.0%) after using jumbo cups^4^, ^8^, ^10^, ^24^. Whaley *et al*. noted that dislocation was the most frequent postoperative complication when jumbo cups were used for revision THA^4^. The dislocation rate in their case series was high (12%). According to Kelley *et al*., there were two possible reasons for a high dislocation rate related to jumbo cups: (i) an extra‐large cup could prevent attachment of soft tissues close to the femoral head; and (oo) an extra‐large cup could lead to impingement of the femur on the acetabular component^26^. In our series, only 1 patient (1.6%) experienced dislocation postoperatively. The main reason for our low dislocation rate may be the use of the large femoral heads. That is, 74.6% (47/63) of our patients were given a femoral head implant with a diameter ≥32 mm, compared with 28.1% (25/89) of those treated by Whaley *et al*.^4^ and 59.3% (64/108) in Lachiewicz and Soileau's study^24^. Patel *et al*. reported a similar lower dislocation rate (4.7%, 2/43) and attributed it to greater anteversion of the acetabular component and the direct lateral surgical approach^9^.

The use of jumbo cups helps bring the COR to a more anatomic (inferior) position, which may improve hip biomechanics^9^. Whaley *et al*. reported 7‐mm lowering of the COR after using jumbo cups during revision hip arthroplasty^4^. Fan *et al*. reported 4‐mm lowering of the COR^10^. In our case series, the COR was improved from 29.7 ± 10.4 mm to 22.3 ± 7.6 mm, similar to those in the abovementioned studies. Using a jumbo cup, however, cannot completely restore an anatomic COR. Based on Nwankwo and Ries's study, the jumbo cup technique resulted in an average COR elevation of approximately 10 mm compared with that on the contralateral side^15^. In the present study, the average COR elevation was approximately 8 mm when using jumbo cups.

There are three limitations to this study. First, errors may occur during the radiographic measurements. These measurements, however, were performed by a single observer in our study. Hence, no intraobserver or interobserver repeatability testing was performed. Second, the operations were performed by several senior surgeons at our institution. Third, we used four different acetabular components in these patients.

### *Conclusions*

The use of jumbo cups during revision THA resulted in excellent mid‐term cup survival, with an acceptable postoperative complication rate. The radiographic measurements in this study showed that the COR could be partly restored when using the jumbo cup technique.
